# Triple therapy combined with ventriculoperitoneal shunts can improve neurological function and shorten hospitalization time in non-HIV cryptococcal meningitis patients with increased intracranial pressure

**DOI:** 10.1186/s12879-020-05510-9

**Published:** 2020-11-16

**Authors:** Min Li, Jia Liu, Xuhui Deng, Qingzhou Gan, Yijie Wang, Xiaofeng Xu, Ying Jiang, Fuhua Peng

**Affiliations:** 1 Department of Neurology, The Third Affiliated Hospital of Sun Yat-Sen University, Guangzhou, 510630 Guangdong China; 2 Department of Neurology, Yuebei People’ Hospital, Shaoguan, 5120264 Guangdong China

**Keywords:** Triple therapy, Ventriculoperitoneal shunts, Non-HIV cryptococcal meningitis, Increased intracranial pressure

## Abstract

**Background:**

Raised intracranial pressure (ICP) and insufficient antifungal regimens are the two main factors result to unsatisfactory outcomes in non-HIV cryptococcal meningitis (CM) patients. In this study, we try to discuss that whether triple therapy of amphotericin B (AmB), fluconazole, 5-flucytosine (5-FC) plus ventriculoperitoneal shunts (VPS) is superior to AmB, 5-FC, fluconazole plus intermittent lumbar puncture in induction therapy in non-HIV CM patients with increased ICP.

**Methods:**

We reviewed 66 clinical records from non-HIV CM patients with increased ICP. The demographic and clinical characteristics, BMRC staging, cerebrospinal fluid profiles (CSF), brain magnetic resonance imaging, treatment, and outcomes of these individuals were retrospectively analyzed. All non-HIV CM patients with increased ICP (≥ 25 cmH2O) were divided into two groups, including 27 patients treated with triple antifungal agents and 39 patients treated with the same triple therapy plus VPS.

**Results:**

Triple therapy plus VPS group had more satisfactory outcomes, more CSF sterilization at 10 weeks follow-up, lower CSF opening pressure, lower BMRC staging scores one week after VPS, less CSF *C. neoformans* counts and CSF culture positive. Besides, these patients had shorter hospital stay than triple therapy group.

**Conclusions:**

Triple antifungal agents combined with VPS could effectively reduce ICP, had faster rate of clearance of *C. neoformans* counts, more improved neurological function, shorten hospitalization time and better outcomes in non-HIV CM patients with increased ICP. Our study indicated that triple therapy plus early VPS may be an optimal treatment for non-HIV CM patients with increased ICP.

## Background

Cryptococcal meningitis (CM) is one of the most common clinical presentations of cryptococcosis, which accounts for more than 223,100 cases and 181,100 deaths per year [1]. Elevated intracranial pressure (ICP) is the most common complication of CM, and can cause impaired mental status, neurological deterioration, and severe disability [2, 3].

The Infectious Diseases Society of America (IDSA) recommended the induction therapy of amphotericin B deoxycholate (AmB) (0.7–1.0 mg/kg per day) combining with 5-flucytosine (5-FC) (100 mg/kg per day) for at least 4 weeks as the preferred regimen for non-HIV-infected and non-transplant patients with CM [4]. However, AmB and 5-FC may often cause severe toxic effects so that CM patients can’t endure the recommended dosages. In addition, increased ICP (≥ 25 cm H_2_O) in CM patients, is associated with short-term survival and impaired treatment response [5]. Raised ICP and only moderately effective antifungal regimens, which frequently take more than 4–6 weeks to sterilize cerebrospinal fluid (CSF), are two main factors that result to unsatisfactory outcomes in CM patients [6]. Despite therapy, mortality rates in HIV-seronegative individuals with CM are high [7]. Our previous study showed that non-HIV patients with triple therapy of AmB, 5-FC plus Fluconazole at sub-therapeutic doses seemed to have a better prognosis than therapy of AmB and Fluconazole [8], and early placement of ventriculoperitoneal shunts (VPS) is helpful in decreasing ICP and fungal overload in non-HIV CM patients [9]. Aggressive management of ICP combined with antifungal therapy is possible the optimal treatment [10]. Therefore, we try to discuss that whether triple therapy of AmB, 5-FC, fluconazole plus VPS is superior to AmB, 5-FC, fluconazole plus intermittent lumbar puncture in induction therapy in non-HIV CM patients with increased ICP.

## Materials and methods

In this study, data were derived from 66 CM patients enrolled from January 2011 to December 2018 at the Third Affiliated Hospital of Sun Yat-sen University, Guangzhou, China. CM patients were identified by symptoms and signs, and positive India ink staining of the CSF or a positive CSF culture for *Cryptococcus neoformans*. All patients were confirmed to be non-HIV infected, with CSF opening pressure ≥ 25 cmH_2_O. These selected CM patients were divided into two groups according to the treatment regimens. In triple therapy group, the 27 patients were treated with a combination of AmB, fluconazole, and 5-FC plus intermittent lumbar puncture. In triple therapy plus VPS group, the 39 patients were administered triple therapy of AmB, fluconazole, and 5-FC plus VPS. Patients meeting one of the following criteria were excluded: (1) prior surgical intervention due to intracranial hypertension; (2) recurrent CM.

The demographic and clinical characteristics, British Medical research council (BMRC) staging [11], CSF profiles, brain magnetic resonance imaging (MRI), treatment, and outcomes of these individuals were analyzed. The BMRC staging system was used to evaluate the severity in this study. We compared the two groups at baseline levels, discharge and follow-up at 10 weeks. The enrolled patients underwent lumbar punctures at least once a week in accordance with guidelines during the implementation of the two study regimens [5, 12]. CSF specimens were routinely evaluated for white blood cell (WBC) counts, glucose, protein, India ink stain and culture. Other conventional blood tests and images were also recoreded during treatment.

The antifungal therapy was initiated as soon as the diagnosis of CM confirmed. In triple therapy group, the patients were treated with intravenous AmB (30–40 mg/d), fluconazole (600–800 mg/d) plus oral 5-FC (4–6 g/d) daily for at least 2 weeks. In triple therapy plus VPS group, the patients received the same triple therapy for at least 2 weeks, and received VPS. The CM treatment response in this study was defined by clinical and mycological criteria [13], particularly the documented clearance of the CSF [14]. The outcome of treatment response was assessed at 10 weeks after the initiation of antifungal therapy. Based on the CSF *cryptococcus* clearance, the therapeutic outcomes were divided as ‘successful response’(including complete response and partial response) or ‘failure’(including stable response, disease progression and death) [15].

Continuous variables (presented as the mean ± standard deviation (SD) or median with ranges) were performed with a t-test or Wilcoxon rank sum test. Categorical variables (expressed as a percentage) were used with Chi-square test, Fisher’s exact test or Wilcoxon rank sum test. 

Significance was set at *P* < 0.05. Laboratory data were analyzed as factors using univariate and multivariate analyses in both groups for the outcome analysis. All statistical analyses were two-sided and performed using SPSS (version 16.0, Chicago, IL, USA) .

## Results

The demographics, baseline CSF parameters, cranial computed tomography (CT)/MRI and baseline BMRC staging of the patients were presented in Table 1. Triple therapy plus VPS group patients had higher burdens of cryptococcal in organisms at baseline (median 9533 vs. 1398 *C. neoformans* counts/ml, respectively, *P* = 0.002), and shorter hospital stay than that of triple therapy group (77 vs. 35, *P* = 0.000). That meant triple antifungal agents combined with VPS could obviously shorten the hospitalization time in non-HIV CM patients with increased ICP (≥ 25 cmH_2_O).

**Table 1:** Baseline patient characteristics

Variables	Group I (AmB+ Fluconazole	Group II (AmB+ Fluconazole	*P*-value
	+ 5-FC,*n* = 27)	+ 5-FC + VPS, *n* = 39)	
Age, years (mean ± SD)	44.1 ± 14.0	47.9 ± 14.7	0.287
Gender, male (n,%)	20 (74.1%)	28 (71.8%)	0.839
Predisposing Factors			
Chronic hepatitis B	5 (18.5%)	4 (10.3%)	0.340
Pulmonary tuberculosis	3 (11.1%)	2 (5.1%)	0.370
Type 2 diabetes mellitus	2 (7.4%)	5 (12.8%)	0.486
Autoimmune diseases	1 (3.7%)	4 (10.3%)	0.326
Solid tumor	1 (3.7%)	4 (10.3%)	0.326
Symptoms duration, days, (med, range)	30 (6–120)	30 (8–90)	0.331
Length of hospital stay days, (med, range)	77 (12–308)	35 (18–73)	0.000^*^
CSF parameters opening pressure			
≥25 cmH2O(n, %)	27 (100%)	42 (93.0%)	0.107
CSF WBC count×106/l (med, range)	92 (10–649)	82 (4–840)	0.744
≥20 (n,%)	23 (85.2%)	32 (82.1%)	0.871
CSF protein g/l (med, range)	0.78 (0.08–2.58)	0.71 (0.22–5.10)	0.579
CSF glucose mmol/l (med, range)	1.19 (0.00–3.79)	0.83 (0.01–3.09)	0.273
CSF cryptococci count/ml (med, range)	1398 (0–57,500)	9533 (0–263,000)	0.002^*^
CSF culture positive (+)	15 (55.69%)	19 (48.7%)	0.114
Brain images (CT or MRI)			
Meningeal enhancement	18 (66.7%)	18 (46.2%)	0.182
Cryptococcosis-related lesions in brain	13 (48.1%)	17 (43.6%)	0.717
Hydrocephalus	3 (11.1%)	3 (7.7%)	0.637
BMRC staging (n, %)			0.443
1	2 (7.4%)	8 (20.5%)	
2	16 (59.3%)	10 (25.6%)	
3	9 (33.3%)	21 (53.8%)	

*AmB* amphotericin B, *5-Fc* flcytosine, *VPS* ventriculoperitoneal shunts, *WBC* white blood cell, *SD* standard deviation, *med* median, *CSF* cerebrospinal fluid, *BMRC* British Medical research council. Data are presented as the mean ± sD, median (range) or n (%). Continuous variables were analyzed by t-test or Wilcoxon rank sum test; categorical variables were analyzed by Chi-square test or Fisher’s exact test.

^*^P < 0.05

The results of CSF sterilization within 10 weeks between the two groups were detailed in Table 2. Compared to triple therapy group, there were more patients in triple therapy plus VPS group to clear *cryptococcus* from the CSF (*P* = 0.033), which suggested that triple antifungal agents plus VPS could more effectively clear cyptococcus from CSF. No significant differences were observed in the early fungicidal activity (CSF sterility within 2 weeks) and persistent infection (CSF sterility beyond 4 weeks [4]) between the two groups.

**Table 2:** Data for the CSF sterility at the 10 weeks follow-up

	Group I (AmB+	Group II (AmB +	*P*-value
	Fluconazole+ 5-FC,*n* = 27)	Fluconazole+ 5-FC + VPS, *n*^a^ = 38)	
case (n, %)	8 (29.6%)	22 (56.4%)	0.033^*^
Days^b^ (mean ± SD)	29.2 ± 20.0	32.5 ± 24.8	0.909
≤ 2 weeks (n, %)	2 (7.4%)	8 (20.5%)	0.662
> 2 weeks (n, %)	6 (22.2%)	14 (35.9%)	
≤ 4 weeks (n, %)	4 (14.8%)	13 (33.3%)	0.730
> 4 weeks (n, %)	4 (14.8%)	9 (23.1%)	

*AmB* amphotericin B, *5-Fc* flucytosine, *Flu* fluconazole, *SD* standard deviation. Continuous variables analyzed by t-test; categorical variables analyzed by chi-square test or Fisher’s exact test

^a^ One patient was lost to -follow-up at the 10th week in Group II

^b^‘Days’ indicates the time from diagnosis to the first negative CSF india ink stain

^*^P < 0.05

The CSF opening pressure, CSF *C. neoformans* counts and CSF culture positive of patients in triple therapy plus VPS group significantly decreased after VPS (*P* = 0.000, *P* = 0.019 and *P* = 0.002, respectively). CSF protein increased significantly after VPS. Patients in triple therapy plus VPS group got the improvement in neurological function circumstances evaluated by comparing the BMRC staging before and after VPS (*P* = 0.026) (Table 3), which suggested that triple antifungal agents combined with VPS could significantly improve neurological function.

**Table 3:** Clinical characteristics of Group II patients before VPS and one weeks after VPS

Patient	Before VPS	one week after VPS	*P*-value
CSF parametersopening pressure			
≥ 25 mH2O(n, %)	33 (84.6%)	12 (30.8%)	0.000^*^
CSF WBC count×106/l (med, range)	66 (4–620)	77 (0–993)	0.220
≥20 (n,%)	36 (80.0%)	41 (91.1%)	0.136
CSF protein g/l (med, range)	0.67 (0.22–4.08)	2.29 (0.08–729)	0.000^*^
CSF glucose mmol/l (med, range)	1.16 (0.01–3.47)	0.91 (0.01–4.86)	0.380
CSF cryptococci count/ml (med, range)	9533 (0–263,000)	2066 (0–235,000)	0.019^*^
CSF culture positive (+) (n, %)	19 (48.7%)	6 (15.4%)	0.002^*^
BMR staging (*n* = 39)			0.026^*^
1	8 (20.5%)	14 (35.9%)	
2	10 (25.6%)	14 (35.9%)	
3	21 (53.8%)	11 (28.2%)	
Complications after VSP (n, %)	NA	20 (44.4%)	NA
Fever 3 days after VPS	NA	16 (35.6%)	NA
Over-shunting	NA	2 (4.4%)	NA
Obstructed shunt	NA	3 (6.7%)	NA
Secondary abdominal infection	NA	1 (2.2%)	NA

*VPS* ventriculoperitoneal shunts, *WBC* white blood cell, *med* median, *CSF* cerebrospinal fluid, *NA* not available. Data are presented as median (range) or n (%). Categorical variables were analyzed by Chi-square test or Fisher’s exact test

^*^P < 0.05

Adverse events were common. Antifungal drugs are associated with toxic effects, particularly AmB and 5-FC. At the end of the 10th week, there were no significant differences in the two groups in objective blood tests (Table 4). Sequelae was mainly headache and cranial nerve deficit (optic and auditory nerves). No significant differences about sequelae in two groups were found.

**Table 4:** Adverse events in objective blood tests during the treatment and sequelae at the 10th week in patients between group I and group II

	Group I (AmB+	Group II (AmB+	*P*-value
	Fluconazole + 5-FC)	Fluconazole + 5-FC + VPS)	
Adverse events (n, %)	(*n* = 27)	(*n* = 39)	
Hypokalemia	13 (48.1%)	20 (51.3%)	0.804
Transaminase elevation	14 (51.9%)	15 (38.5%)	0.285
Renal impairment	5 (18.5%)	12 (30.8%)	0.267
Hematological impairment	5 (18.5%)	14 (35.9%)	0.128
Sequelae	(*n* = 27)	(*n*^a^ = 36)	
Headache	9 (33.3%)	9 (25.0%)	0.395
Dizziness/vertigo	2 (7.4%)	5 (13.9%)	0.465
Hemiparesis	4 (14.8%)	5 (13.9%)	0.850
Visual	8 (29.6%)	6 (16.7%)	0.184
Auditory	1 (3.7%)	7 (19.4%)	0.077
Seizure	3 (11.1%)	2 (5.6%)	0.387

^a^ One patient was lost to -follow-up and two patients were death at the 10th week in Group II

In triple therapy plus VPS group, after the VPS, thirteen cases had low to moderate fever, and the temperature recovered to normal in a week by using antibiotics. One case occured shunt obstruction and he was taken second operation on the other side. One case turned up excessive shunt after operation, and his CSF open pressure was normal by adjust the drainage tube. There was no cryptococcal infection spread to other organs (Table 3).

**Fig. 1:**
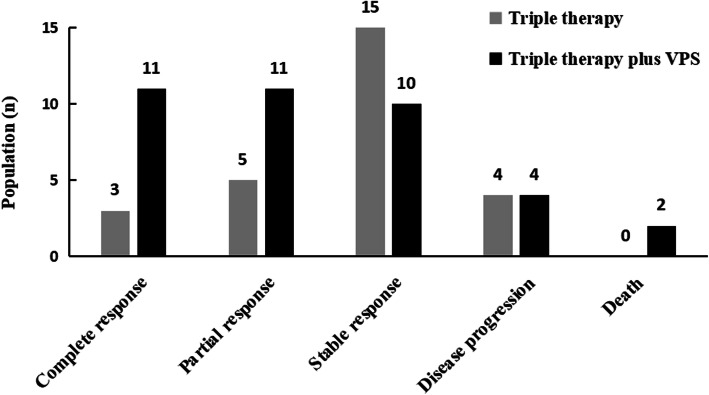
Treatment outcomes according to induction therapy at the 10th week after the initial therapy

**Fig. 2:**
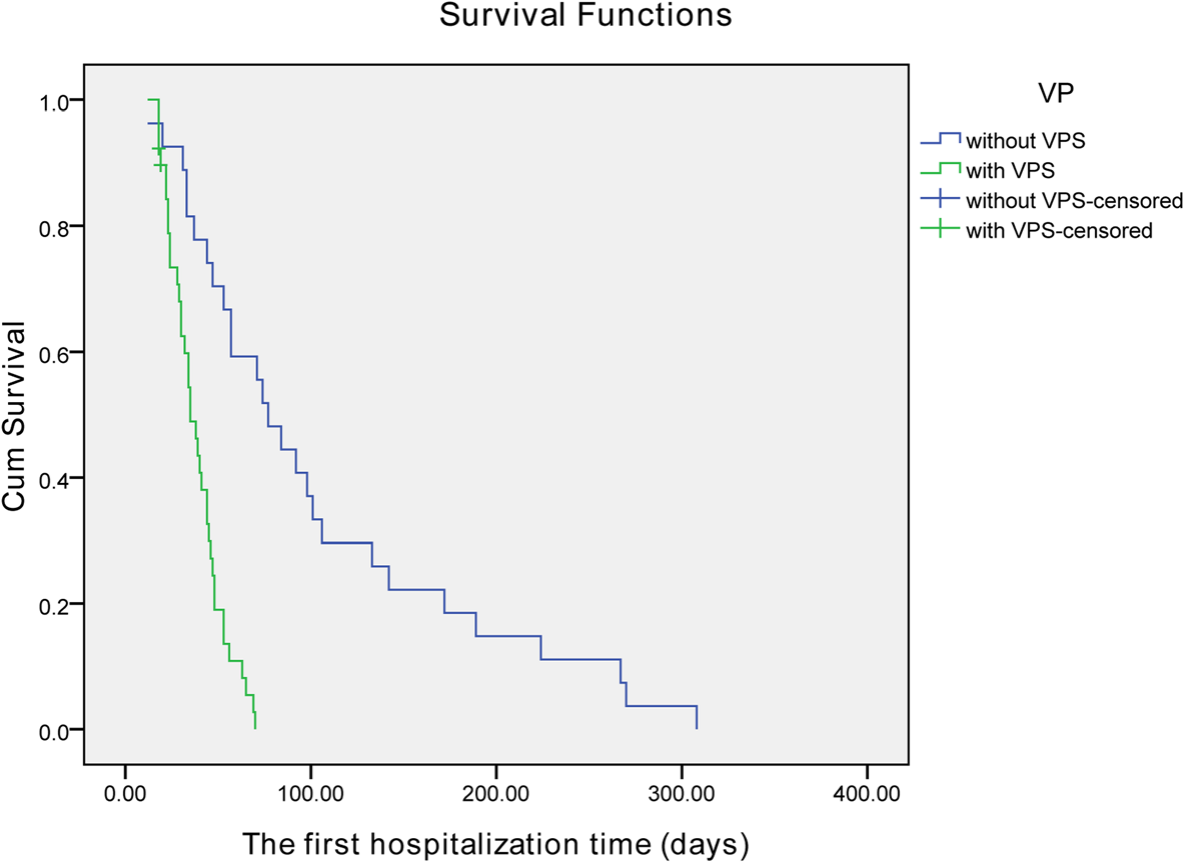
Kaplan–Meier survival curves of triple therapy plus VPS group and triple therapy group

The primary outcome of treatment response was evaluated at the 10th week after the initial therapy. The total ‘successful response’ rate (including complete and partial responses) in triple therapy plus VPS group reached 57.9% (22/38), whereas the rate was 29.6% (8/27) in triple therapy group (*P* = 0.025) (Table 5 and Fig. 1). Two patients in triple therapy plus VPS group died of multiple organ failure on the 18st day and 47st day respectively. One patient died in triple therapy group after the 10 weeks. Triple therapy plus VPS group patients have shorter the first hospitalization stay than triple therapy group (Fig. 2, log Rank p<0.000). In the multivariate analysis, we did not find meaningful predictors of an satisfactory outcome in the triple therapy group. The multivariate analysis showed high red blood cells levels (OR 3.210, 95% CI 1.097–9.396; *P* = 0.033) to be a good prognosic factor for the patients with triple therapy plus VPS (Table 6).

**Table 5:** Treatment outcomes in patients between group I and group II at 10 weeks

	Group I (AmB+	Group II (AmB+	*P*-value
	Fluconazole + 5-FC,*n* = 27)	Fluconazole + 5-FC + VPS, *n* = 39)	
Lost to follow-up	0 (0.0%)	1 (2.3%)	0.399
Successful response	8 (29.6%)	22 (57.9%)	0.025^*^
Complete response	3 (11.1%)	11 (28.9%)	
Partial response	5 (18.5%)	11 (28.9%)	
Failure	19 (70.4%)	16 (42.1%)	0.350
Stable response	15 (55.6%)	10 (26.3%)	
Disease progression	4 (14.8%)	4 (10.5%)	
Death	0 (0.0%)	2 (5.3%)	

^*^p < 0.05

**Table 6:** Factors associated with satisfactory outcomes after treatment of triple therapy group and triple therapy plus VPS group

Variables	Univariate analysis			Multivariate analysis		
	OR	95% CI	*p*-Value	OR	95% CI	*p*-Value
Group I						
CSF protein	4.924	0.855–28.354	0.074			
CSF culture	9.625	0.980–94.540	0.052			
Group II						
Red blood cells	2.880	1.043–7.957	0.041	3.210	1.097–9.396	0.033
Blood creatinine	1.046	1.000–1.094	0.050			

## Discussion

In this study, we found that triple antifungal agents combined with VPS could effectively reduce ICP, had faster rate of clearance of Cryptococcus, more improved neurological function, shorten hospitalization time and better outcomes in non-HIV CM patients with elevated ICP (≥ 25 cmH_2_O) than triple therapy group. No significant differences were observed in the incidence of adverse events and sequelae between the two groups.

As stated in our previous study [8], there were several reasons for patients treating with triple therapy. Patients could not bear AmB and 5-FC at the recommended doses from the beginning and (or) could not be maintained at the effective dose during induction therapy because of drug toxic effects [4, 16]. Previous research had suggested early mycological clearance was favored with AmB deoxycholate plus fluconazole combination therapy [17]. Although AmB and fluconazole are theoretically antagonistic drugs, in vitro and clinical observations support the effectiveness of the combination [6, 18, 19]. Fluconazole in triple therapy administered at a conventional dosage may have compensated for the less role of AmB at lower dosage, particularly when fluconazole is combined with 5-FC [20]. Patients enrolled in this study were treated with triple therapy at conventional or sub-therapeutic doses for the induction treatment. And no significant adverse events were found in objective blood tests during the treatment between two groups.

Elevated ICP is an important risk factor for high mortality and poor outcomes in CM patients [21, 22]. Among patients with uncontrollable increased ICP, the best way to decrease the pressure is to implant a permanent shunt [23]. VPS can effectively reduce ICP. The complications after VPS were controllable. Neurological function assessment by BMRC stage, was significantly improved after VPS. But the sequelae of the triple therapy plus VPS group wasn’t significantly less than the triple therapy group. We proposed that early placement of a VPS is helpful in preventing irreversible neurological complications. Increased ICP (≥ 25 cmH_2_O) is a severe complication in patients with CM, and is generally considered to relate to high fungal burden in the CSF and high mortality in CM [6, 24, 25]. A negative CSF culture at the end of the induction phase is an important predictor of fungal clearance at 10th week [26]. The goal of CM with ICP treatment is to reduce fungal burden quickly and to prevent long-term neurological deficits. In this study, triple therapy plus VPS group had faster rate of clearance of Cryptococcus than triple therapy group. Hung CW et al. reported that the mean hospitalization duration was 74.7 days and 70.6 days and interval between meningitis onset to shunting procedure was 68.7 days and 38.7 days for CM patients placed VPS with and without CSF over drainage, respectively [27]. Triple therapy group in our study had the similar result of hospital stay 77 days. Hospital stay of triple therapy plus VPS group was significantly shortened (35 days), which may be result of triple antifungal agents and early VPS. CM patients with elevated ICP who placing a VPS could be explained for following factors. CM patients had a typical presentation with substantially increased ICP along with severe headaches, nausea and vomiting, loss of consciousness, marked visual and auditory changes. Patients responded poorly for intermittent lumbar puncture to decrease the ICP or required continuous lumbar CSF drainage to remain neurologically asymptomatic. Patients in triple therapy plus VPS group mostly emerged aggravated symptoms or new symptoms in a short time before VPS. In addition, the timing and choice of VPS placement may be related to preferences of neurosurgery or physicians [28]. Recent study indicated that early shunt surgery is mandatory for the treatment of CM complicated by hydrocephalus and/or increased ICP to avoid irreversible neurological complications [27]. In our study, patients received VPS after admission with triple therapy, with lower CSF pressure, faster reduction of CSF *C. neoformans* counts. Baddley et al.'s study also suggested that earlier permanent VPS placement and more aggressive treatment may potentially improve outcomes for CM patients with increased ICP [28]. Treatment in triple therapy plus VPS group decreased the CSF pressure, CSF *C. neoformans* counts quickly, and certainly resulted to more ‘successful response’ rate (including complete and partial responses) than that in triple therapy group at 10 weeks follow-up.

Our study was performed in a single hospital by retrospective review of the medical records. The number of patients enrolled was small, and the dosage range of the three antifungal drugs was insufficient. Large multicenter real-life studies are needed to further verify the efficiency and feasibility of this method.

## Conclusions

In summary, based our previous work, triple antifungal agents combined with VPS group could effectively reduce ICP, had faster rate of clearance of Cryptococcus, more improved nerve function, shorten hospitalization time and better outcomes in non-HIV CM patients with elevated ICP than triple therapy group. It is an optimal treatment with aggressive management of intracranial pressure and induction therapy with antifungal combination in non-HIV CM patients with intracranial hypertension (≥ 25 cmH_2_O).

## Data Availability

The data and materials used during the current study are available from the first author on reasonable request.

## Abbreviations

ICP: Intracranial pressure
CM: Cryptococcal meningitis
AmB: Amphotericin B
5-FC: 5-flucytosine
CSF: Cerebrospinal fluid Profiles
CT: Computed tomography
MRI: Magnetic resonance imaging
BMRC: British Medical Research Council
IDSA: Infectious Diseases Society of America
med: Median
SD: Standard deviation
VPS: Ventriculoperitoneal shunts
WBC: White blood cell
